# Recording Medical Errors in Brain Tumor Surgery Can Facilitate Their Avoidance: Single Institution Comparative Cohort Analysis over 19 Years

**DOI:** 10.3390/curroncol33050281

**Published:** 2026-05-09

**Authors:** Mohammed J. Asha, Patrick E. Steadman, Ashkan Samienezhad, Mark Bernstein

**Affiliations:** Division of Neurosurgery, Toronto Western Hospital, University Health Network (UHN), Toronto, ON M5T 2S8, Canada; mohammedjamil80@yahoo.com (M.J.A.); patrick.steadman@mail.utoronto.ca (P.E.S.); ashkan.samienezhad@gmail.com (A.S.)

**Keywords:** adverse events, complications, medical errors, neurosurgery, patient safety, quality improvement

## Abstract

**Objectives**: Error-tracking studies have become an invaluable tool for gaining deeper insights into the patterns, causes, and clinical consequences of errors. In this paper, we examine the long-term impact of analyzing errors at the level of an individual surgeon. **Methods**: We performed a retrospective analysis of an internally maintained prospective database from September 2013 to April 2019 (Group C). These variables were compared with two earlier cohorts: Group A (2000–2006) and Group B (2006–2013). The key endpoints assessed included error incidence, type, severity, preventability, and clinical impact. The study also investigated the potential seasonal variations in error incidence, considering the rotation of trainees. **Results**: A persistent decrease in the mean errors/case (across all types) since records began in 2000 was noted; (1.8 for study group C versus 1.9 and 2.4 for group B and group A respectively, *p* = 0.048). We observed a drop in error severity score (major errors of 17.5% versus 29.5% and 22.6% respectively, *p* < 0.00001) and clinical impact (high impact in 0.4% versus 1.0% and 2.7% respectively, *p* < 0.00001). **Conclusions**: Ongoing and systematic error tracking and analysis seem to have a lasting impact on reducing both the incidence and severity of errors. These positive outcomes are thought to reflect the establishment of a culture of safety, fostering transparency and constructive feedback within the broader surgical team.

## Simple Summary

Medical errors are a critical concern in surgery, and more broadly in healthcare. We examined the long-term impact of a surgeon-specific error-tracking system over nearly twenty years. Our findings reveal a significant, steady decline in the number and severity of surgical errors over time. Our research shows that systematic tracking fosters a culture of transparency and safety. By providing evidence that long-term monitoring works, our study encourages others to adopt similar data-driven approaches. We also show tracking helps surgical teams better manage the risks associated with the transitions of new trainees into the clinical environment.

## 1. Introduction

Advances in the study of medical errors were built upon two elements of healthcare. The first is the recognition that healthcare providers at all levels are human, and the second is that many errors are the result of systematic rather than individualistic deficiencies. Error-tracking studies have become an invaluable tool for gaining deeper insights into the patterns, causes, and clinical consequences of errors [1,2,3]. The aviation industry was a pioneer in implementing comprehensive measures to study, record, and prevent errors, leading to the development of crisis management training and Crew Resource Management (CRM) systems [4,5,6]. While the medical field was initially slow to adopt similar approaches, such paradigms were eventually incorporated into medical training to mitigate the effects of errors, with anaesthesiologists being among the earliest adopters [7]. The Harvard Medical Practice study is often regarded as one of the first to investigate this issue [8], but it was the release of the Institute of Medicine (IOM)’s report that truly brought the problem of patient harm due to medical errors to the forefront of both professional and public concern [2].

Public awareness of healthcare safety issues has grown, driven by the recognition that medical errors come at a significant cost, both economically and, and more importantly through human suffering, morbidity, and mortality. Medical errors are estimated to cause between 44,000 and 98,000 deaths annually in the United States [1,9]. The IOM report placed the economic burden of these errors at an estimated $17–29 billion per year [2]. Despite this, it is only in recent years that studies on self-reported medical errors have emerged [10,11]. This shift in medical practice has been accompanied by the implementation of safety measures such as safety checklists for surgeries, as well as initiatives like e-prescriptions, automated ordering systems, and built-in electronic warning systems to enhance patient safety [10,12,13].

Neurosurgery is among the specialties most prone to medical errors [8,14], yet it is argued that errors in this field are often under-examined. This may stem from the misconception that in such a precise and delicate specialty, errors are unacceptable or even unethical [11]. Such beliefs not only contribute to the public’s unrealistic expectation that medical professionals should be infallible and that all errors are due to negligence, but they also place a considerable psychological burden on healthcare providers [15,16,17].

Over recent decades, patients’ perceptions of medical errors have evolved. Although initial concerns about medico-legal repercussions were prevalent, numerous studies have shown that patients are more likely to understand and accept medical errors when they are disclosed honestly and transparently [1,11,18]. This shift, combined with improved safety measures to prevent or mitigate the effects of medical errors, may help explain the rise of defensive medicine and the relative decline in medico-legal claims [19].

Our study reflects nearly 20 years of researching errors in neurosurgery, contained partially in two prior cohort studies [14,20]. We previously reported a reduction in mean number of errors from 2000 to 2013; however, this was contrasted with a trend toward increasing severity scores for near-misses [20]. At that time, the shift was attributed to the development of a culture of awareness, where proactive error identification, analysis, and discussion led to their prevention. However, it remains unclear if this eventually led to a reduction in severe near-misses.

To assess the validity and long-term sustainability of this approach, we conducted a comparative cohort analysis covering the period from 2013 to 2019. Additionally, we explored the possibility that, in large teaching neurosurgical units with frequent rotations of residents and fellows, there might be a seasonal pattern of errors linked to the arrival of new or less experienced trainee surgeons. We examine whether effects observed in prior studies have yielded meaningful benefit to patients with reductions in error incidence and severity.

## 2. Materials and Methods

All errors (defined as any deviation from perfection) that occurred in the perioperative period on all electively scheduled surgeries were prospectively recorded immediately following the conclusion of every procedure and as needed until discharge in a dedicated and self-maintained database by the senior author. The period for capturing these errors started from the moment the patient was called to the OR until they were discharged from hospital. The inclusion criteria were: operation performed under the supervision of the senior author, and elective procedure within regular day-time hours and with complete medical records and a dataset on the error database. All detected errors were either observed directly by the senior author or reported to him. The senior author entered all data on his prospectively maintained database.

The recorded variables included: patient demographics, surgical procedure, type of anaesthesia, American Society of Anesthesiologists (ASA) score, the presence or lack of medical errors, along with the number of errors per case, their severity score, their preventability score, as well as any recorded clinical complications and whether they were related to the recorded errors or not. These variables were defined according to the previously adopted definitions (definitions in Table 1) [14] allowing for longitudinal monitoring.

The study period for Cohort C extended from September 2013–April 2019. The period ends due to change in practice by the senior author. Only elective procedures performed within regular hours were included in this analysis. In total 95% of the cases were neuro-oncology cases. Cohorts A (2000–2006) and B (2006–2013) were previously published components of the database [14,20] of distinct 6–6.5 year periods. In Cohort C, the surgical practice of the senior author changed, where they stopped performing elective spinal procedures in late 2012.

To analyze seasonal variations related to clinical fellows’ and resident’s rotations, cases were clustered according to the month when surgery was performed and all errors committed in the same month of every year were included. The annual variation analysis included all errors recorded in each calendar year. Cohort analysis against the two previously reported cohorts followed the previously reported parameters.

All baseline characteristics and comparative analyses were standardized using patient-level denominators across all cohorts to ensure internal consistency.

Statistical analysis was done using IBM SPSS 22 software (IBM Corp., Armonk, NY, USA). Continuous variables are presented as mean ± standard deviation and were compared across cohorts using one-way analysis of variance (ANOVA). For categorical data, a Chi Square Test or Fisher’s exact tests were used. Given the longitudinal and observational nature of the dataset, statistical comparisons are interpreted as exploratory and hypothesis-generating rather than confirmatory. The level of statistical significance was defined as *p* value < 0.05. Research Ethics Board approval from the University Health Network was obtained. The REB approval study number 19-5064.0 dated 22 January 2019.

## 3. Results

**Figure 1:**
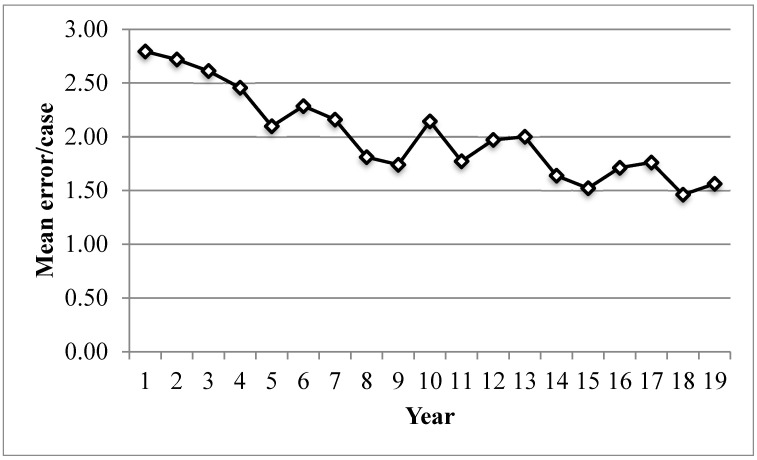
Mean error/case plotted against timeline from 2000 to 2018 (2019: Jan to April only).

The total number of performed cases for Cohort C was 684 (Table 2). We found a mean error per case of 1.8. There was a progressive reduction in mean errors per case across the three cohorts (2.4 vs. 1.9 vs. 1.8, *p* = 0.048, Figure 1).

The distribution of procedure type differed significantly between cohorts (*p* < 0.00001), reflecting the evolution of surgical practice toward predominantly cranial neuro-oncological procedures. Similarly, the distribution of anaesthesia type differed significantly across cohorts (*p* < 0.00001). Mean ASA score increased across cohorts (*p* < 0.0001), suggesting a trend toward higher patient complexity over time.

Detailed analysis of medical errors for this period compared to the two previous cohorts is presented in Table 3. Across most categories the number of errors decreased. We found technical, management/judgement and communication errors decreased (*p* < 0.0001, *p* < 0.045 and *p* < 0.0019 respectively). The proportion of major errors decreased significantly over time (22.6% vs. 29.5% vs. 17.5%, *p* < 0.00001), accompanied by a marked reduction in high clinical impact errors (2.7% vs. 1.0% vs. 0.4%, *p* < 0.00001). These findings demonstrate a consistent improvement in both error frequency and clinical severity.

**Figure 2:**
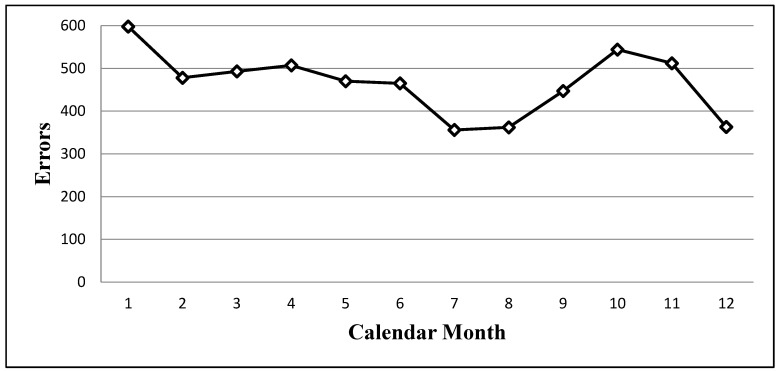
Number of clustered according to calendar months cumulatively for the period 2000–2019.

The calendar month across the three cohorts showed variation in error number (Figure 2). The lowest number of errors were in May–July, which marks the end of the training year for clinical fellows and most residents (*p* > 0.05).

## 4. Discussion

Our previous work has demonstrated the positive impact of ongoing error recording. Between 2006 and 2013, we observed a significant reduction in the total number of errors compared to the previous cohort (2000–2006), with the mean error rate decreasing from 2.4 errors per case to 1.9 errors per case [20]. In the current cohort (2013–2019), the mean error rate is 1.8 errors per case, reinforcing the lasting benefits of this approach. We also report the case mix moved towards cranial, and the mean ASA increased with cohorts; these differences are not corrected for across cohort mean error rates. Though typically higher ASA results in higher case complexity. Overall, our findings, descriptive as cohort differences exist, are also in line with other institutions, as supported by a prospective study finding error rates of 1.5 errors/case [21].

Our earlier report also found that while the number of errors and their clinical impact (i.e., complications) decreased significantly, the severity score for errors increased, likely due to a rise in near-misses. This suggests that the study of errors helped prevent major clinical complications by transforming them into less harmful near-misses [20]. In the current cohort, however, the reduction in errors was accompanied by a parallel decrease in error severity and clinical complications (Table 3), a trend that suggests incremental improvements in clinical practice. This is further supported by a decrease in errors attributable to anesthesia and nursing, signifying improved team performance. These overall improvements suggest the cumulative experience and knowledge gained by the team enable them to better anticipate and intercept errors before they cause harm.

Our dedicated, concurrent error reporting and analysis system provides a robust and effective mechanism for studying errors. By focusing on error patterns, severity, preventability, and clinical impact, these systems have been shown to significantly reduce errors and improve care quality [22,23,24]. This approach not only improves error detection at the individual or departmental level but also emphasizes identifying system failures rather than assigning blame to individuals [25]. The long-term benefits of such an approach are increasingly recognized in the medical community.

Our strategy is complimentary to traditional morbidity and mortality (M&M) rounds, also known as quality assurance or improvement rounds. These rounds provide valuable insights into lessons learned, offering educational opportunities for residents and fellows, and facilitating team discussions. When structured effectively, with consistent senior clinician participation and a robust data collection system, these rounds can significantly contribute to patient safety and quality improvement [26,27]. However, they have been criticized for their retrospective nature, which can limit their effectiveness in preventing errors. They furthermore often suffer from inconsistent processes, ineffective data collection, and reluctance among staff to openly discuss errors due to fears of punitive consequences [26,28]. Furthermore, traditional M&M reviews tend to focus on diagnostic oversights and often fail to capture in-hospital, post-discharge complications, and near-misses [28,29,30,31]. We believe our prospective medical error tracking can provide an avenue to overcome some of the limitations in M&M rounds.

The clinical impact of medical errors in neurosurgery can be catastrophic, often leading to significant morbidity, mortality, and prolonged patient suffering [12]. Beyond the tragic human cost, the financial burden on the healthcare system is immense, stemming from extended hospital stays, increased resource use, secondary interventions, loss of productivity, and the costs associated with medical litigation and compensation [2,22,32,33]. Additionally, medical errors not only erode public trust in healthcare professionals but can also undermine the confidence of medical practitioners themselves, which can have equally damaging effects on patient care and the well-being of healthcare staff. Institutional solutions to addressing medical errors should then be twofold, combining M&M rounds with prospective error tracking to help reduce systemic causes of medical errors.

An analysis of seasonal variations in error rates did not reveal any significant patterns to suggest that the influx of rotating trainees or fellows contributed to an increase in errors. In fact, there was a slight trend toward fewer errors at the end of the training year (May-August), which may reflect the accumulated experience of junior surgeons as they become more familiar with the surgical environment and protocols. Furthermore, a culture of safety can help trainees learn from adverse effects and navigate complications [34]. Error tracking is a tool for systemic quality improvement, it may also provide an education tool similar to the role M&M rounds serve [28].

The positive outcomes observed in this study may be attributed to the intentional cultivation of a safety culture within the surgical team. By fostering an environment built on confidence, honesty, and transparency, both among team members and with patients, the focus shifts from individual blame to collective responsibility. This culture of openness encourages early recognition and interception of errors, ultimately promoting a proactive approach to error prevention. Instead of assigning fault to individuals, attention is directed toward identifying systemic failures, which appears to be a more effective and sustainable strategy for improving patient safety and minimizing errors in the long term.

## 5. Limitations

The study design is subject to several limitations. The study is based on a single-surgeon prospectively maintained dataset, which may introduce observer and reporting bias. Internally it does not have a second rater or assessor of errors. However, the consistent methodology applied over 19 years provides a degree of internal validity for longitudinal comparisons. Furthermore, the use of arbitrary (non-validated) scales for error severity and preventability, and observer bias are inherent limitations to our study design. Given this study is retrospective in design there are no means of error external validation. Cases included are limited to elective procedures and thus the effect of emergent cases are not included in our analysis, which may have a different error rate. The retrospective comparative analysis is subject to inherent limitations, including evolving case-mix and potential unmeasured confounders. These are not corrected for in our descriptive analysis. Additionally, aspects of the dataset span nearly two decades, during which data recording systems evolved. While the structured dataset used for analysis remains robust, some earlier data elements were archived in formats that required standardization during analysis. Another limitation is the lack of widespread adoption of this error-recording system, which should caution any broader implementation until other institutions perform similar studies. This however is not a new challenge or one unique to this institution [3].

The interpretation of statistical findings in this study must be considered in the context of our longitudinal observational design. While statistically significant associations were identified, these reflect evolving patterns within clinical practice rather than causal relationships. The observed improvements represent the combined effects of increased surgical experience, enhanced team dynamics, and the sustained impact of prospective error awareness.

## 6. Conclusions

This study highlights the significant and lasting benefits of systematically recording and analysing errors at the individual neuro-oncological surgeon level, with the potential for these practices to be applied more broadly at the institutional level. The observed improvements in error reduction, particularly those with clinical impact, suggest a clear learning curve associated with error analysis by a single surgeon. However, this effect appears less pronounced for near-misses, which may require a higher level of awareness and proactive engagement to anticipate and prevent errors, even in the absence of clinical harm. These findings support the role of structured, prospective error tracking as a sustainable strategy for improving surgical safety within complex clinical environments by a single surgeon. We believe that this study, along with its predecessors, provides a solid foundation for future research aimed at standardizing, validating, and expanding the study of medical errors in surgery.

## Figures and Tables

**Table 1:** Definitions of error nomenclature.

Word or Phrase Definition	
error	any act of omission or commission resulting in deviation from a perfect course for the patient; a perfect course was defined as one in which nothing went wrong, from the smallest detail (such as dropping a sponge) to the most obvious example (that is, one that every neurosurgeon would easily recognize) †
adverse event/complication	unintended result of medical treatment that results in prolonged hospital stay, morbidity, or mortality; it may also be an injury caused by medical management rather than the underlying condition of the patient
Type of Error †	
technical	problems in the use of correctly functioning equipment or the performance of an appropriate procedure (for example, aspirator inadvertently bruised brain)
contamination	e.g., instrument required re-sterilization
equipment failure or missing	e.g., instrument required re-sterilization
delay	e.g., long wait for a spine-localizing radiograph
nursing	e.g., nurse failed to properly set up piece of equipment
anesthesia	e.g., anesthetist prematurely extubated patient requiring urgent re-intubation
management/judgment	e.g., patient arrived in operating room w/an abnormal blood result missed by the team
communication/information	e.g., no prophylactic antibiotics administered because the anesthetist did not hear the surgeon’s request
Characteristics of Error Severity †	
major	actual or potential (near-miss) nature of error to cause complication caused actual or potential morbidity or mortality
minor	did not cause actual or potential morbidity or mortality
Clinical Impact of Error † ‡	
clinical impact of error †	deals w/actual nature (impact) of errors in a more explicit way; ability of errors to cause potential problems (near-miss errors) not considered
none	self-explanatory
minimal	self-explanatory
transient	self-explanatory
permanent	self-explanatory
death	self-explanatory
preventability of error † Low (scores of 1–5) deemed non-preventable. High (scores of 6–10) deemed preventable	

† Definitions of terms—adopted from [20]. ‡ Deals with actual nature (impact) of errors in an explicit way; ability of errors to cause potential problems (near-miss errors) not considered.

**Table 2:** Baseline characteristics of cohort (2013–2019).

Variable	Cohort A		Cohort B		Cohort C		*p* Value
Number patients	1108		974		684		
Mean/case ± std	2.4 ± 1.76		1.9 ± 1.46		1.8 ± 1.30		0.048 †
Type of procedure							
Cranial	843	76.1%	875	89.8%	658	96.2%	<0.00001 *
Spinal	252	22.7%	90	9.2%	0	0.0%	
Other	13	1.2%	9	0.9%	26	3.8%	
Anesthesia							
GA	750	67.7%	550	56.5%	399	58.3%	<0.00001 *
Local w or w/out neuroleptic	358	32.3%	414	42.5%	285	41.7%	
Mean ASA	2.41 ± 0.69		2.82 ± 0.59		3.00 ± 0.46		<0.0001 †

* Chi Square Test. † One-way ANOVA.

**Table 3:** Cohorts’ comparison: Cohort A represents cases (2000–2006), Cohort B represents (2006–2013) and Cohort C represents (2013–2019).

Error Type	Cohort A		Cohort B		Cohort C		*p* Value *
Technical	747	27.83%	352	18.60%	232	18.46%	<0.00001
Contamination	678	25.26%	431	22.78%	335	26.59%	0.028
Equipment	489	18.22%	538	28.44%	269	21.32%	<0.00001
Delay	336	12.52%	335	17.71%	316	25.06%	<0.00001
Nursing	152	5.66%	61	3.22%	16	1.27%	<0.00001
Anesthesia	119	4.43%	73	3.86%	21	1.65%	<0.00001
Management/judgment	76	2.83%	51	2.70%	21	1.65%	0.045
Communication/info	51	1.90%	17	0.90%	10	0.82%	0.002
Other	36	1.34%	34	1.80%	40	3.17%	0.0001
Error Characteristics							
Severity							
Major	606	22.58%	558	29.49%	220	17.45%	<0.00001
Minor	2077	77.38%	1329	70.24%	1019	80.90%	
Missing	1	0.04%	5	0.26%	21	1.65%	
Clinical impact							
Low ^	2611	97.3%	1873	99%	1255	99.6%	<0.00001
High §	73	2.7%	19	1.0%	5	0.4%	
Preventability							
Low	578	21.5%	269	14.2%	184	14.6%	<0.00001
High	2106	78.5%	1623	85.8%	1076	85.4%	

* *p* value from Chi Square Test or Fisher’s Exact test. ^ Low impact included three groups (no impact, minimal impact and transient). § High impact included two groups (permanent impact and death).

## Data Availability

The data presented in this study are available on request from the author M.J.A. or M.B.

## Abbreviations

The following abbreviations are used in this manuscript:

CRM	Crew Resource Management
IOM	Institute of Medicine
ASA	American Society of Anesthesiologists
M&M	Morbidity and Mortality
